# Rs868058 in the Homeobox Gene *HLX* Contributes to Early-Onset Fetal Growth Restriction

**DOI:** 10.3390/biology11030447

**Published:** 2022-03-16

**Authors:** Wioletta Izabela Wujcicka, Marian Kacerovsky, Michał Krekora, Piotr Kaczmarek, Beata Leśniczak, Mariusz Grzesiak

**Affiliations:** 1 Scientific Laboratory of the Center of Medical Laboratory Diagnostics and Screening, Polish Mother’s Memorial Hospital-Research Institute, 93-338 Lodz, Poland; 2 Department of Obstetrics and Gynecology, University Hospital Hradec Kralove, Charles University, 500 03 Hradec Kralove, Czech Republic; marian.kacerovsky@gmail.com; 3 Biomedical Research Center, University Hospital Hradec Kralove, 500 03 Hradec Kralove, Czech Republic; 4 Department of Obstetrics and Gynecology, Polish Mother’s Memorial Hospital-Research Institute, 93-338 Lodz, Poland; krekoram@poczta.onet.pl; 5 Department of Gynecology and Obstetrics, Medical University of Lodz, 93-338 Lodz, Poland; mariusz.grzesiak@gmail.com; 6 Laboratory of Prenatal Fetal and Maternal Diagnostics, Polish Mother’s Memorial Hospital-Research Institute, 93-338 Lodz, Poland; kaczmarekpiotr1@gmail.com; 7 2nd Department of Gynecology and Obstetrics, Medical University of Lodz, 90-251 Lodz, Poland; beata.lesniczak@umed.lodz.pl; 8 Department of Perinatology, Obstetrics and Gynecology, Polish Mother’s Memorial Hospital-Research Institute, 93-338 Lodz, Poland

**Keywords:** pregnancy, FGR, SNP, *HLX*, homeobox genes, angiogenesis

## Abstract

**Simple Summary:**

Fetuses with hypotrophy (FGR, fetal growth restriction) are too small for their gestational age and may be prone to various diseases and loss of life. This study aimed to determine the role of single nucleotide polymorphisms (SNPs), located in two homeotic and two angiogenesis-related genes, in the occurrence of FGR, by analyzing blood samples from 380 women in singleton pregnancies. We found that the AT heterozygotes in *HLX* rs868058 were significantly associated with an approximately two-fold increased risk of FGR, diagnosed before 32 weeks of gestation (early-onset FGR). AT heterozygotes were significantly more frequent in women with early-onset FGR than in those with late-onset FGR (diagnosed from 32 weeks of gestation) and compared with healthy subjects. In conclusion, the AT genotype in *HLX* rs868058 may be a significant risk factor for the development of early-onset FGR. So far, the only therapeutic strategy for the management of early-onset FGR is to monitor and terminate pregnancy when the risk of fetal immaturity is lower than the risk of intrauterine death. Therefore, the disclosure of the mechanisms of action of the heterozygous AT state in *HLX* rs868058 would be important to identify plausible targets for new therapeutic approaches to treat the condition.

**Abstract:**

Fetal growth restriction (FGR) is a condition that characterizes fetuses as too small for their gestational age, with an estimated fetal weight (EFW) below the 10th percentile and abnormal Doppler parameters and/or with EFW below the 3rd percentile. We designed our study to demonstrate the contribution of single nucleotide polymorphisms (SNPs) from *DLX3* (rs11656951, rs2278163, and rs10459948), *HLX* (rs2184658, and 868058), *ANGPT2* (−35 G > C), and *ITGAV* (rs3911238, and rs3768777) genes in maternal blood in FGR. A cohort of 380 women with singleton pregnancies consisted of 190 pregnancies with FGR and 190 healthy full-term controls. A comparison of the pregnancies with an early-onset FGR and healthy subjects showed that the AT heterozygotes in *HLX* rs868058 were significantly associated with an approximately two-fold increase in disease risk (*p* ≤ 0.050). The AT heterozygotes in rs868058 were significantly more frequent in the cases with early-onset FGR than in late-onset FGR in the overdominant model (OR 2.08 95% CI 1.11–3.89, *p* = 0.022), and after being adjusted by anemia, in the codominant model (OR 2.45 95% CI 1.23–4.90, *p* = 0.034). In conclusion, the heterozygous AT genotype in *HLX* rs868058 can be considered a significant risk factor for the development of early-onset FGR, regardless of adverse pregnancy outcomes in women.

## 1. Introduction

Fetal growth restriction (FGR, fetal hypotrophy) is a condition that characterizes fetuses as too small for their gestational age that reveal an estimated fetal weight (EFW) below the 10th percentile and abnormal Doppler parameters and/or with EFW below the 3rd percentile, diagnosed in approximately 3–10% of all pregnancies [1,2]. FGR results from impaired genetic growth potential due to a pathological process of various etiologies, which leads to hypoxia and malnutrition of the fetus, imposing a serious threat to its health and life [3]. An FGR-affected newborn may be unable to maintain normal body temperature and may present respiratory distress, hypo- or hyperglycemia, susceptibility to infections, as well as cognitive delays plus neurological and psychiatric disorders in childhood [4,5,6].

Homeotic (homeobox) genes are the most important transcription factors that play a fundamental role in body structure pattern formation [7]. Several homeotic genes have been reported to be involved in placenta and embryo development [2,7]. In mouse models, the disrupted functions of distal-less homeobox 3 (*Dlx3*), extraembryonic, spermatogenesis, homeobox 1 (*Esx1*) and TGFB-induced factor homeobox 1 (*Tgif1*) genes resulted in an abnormal placenta development and embryonic hypotrophy [8,9]. In women with idiopathic FGR, significant differences were noted in the expression of the *DLX3* and H2.0-like homeobox (*HLX*) genes, as well as in *HLX* downstream targets, compared to healthy controls without fetal hypotrophy [10,11,12]. Reduced expressions of *HLX* and ESX homeobox 1 (*ESX1L*) genes were found in a placenta with idiopathic FGR compared to those from control pregnancies [11]. On the other hand, in case of the *DLX3*, *DLX4* and *TGIF1* genes, an increased expression was shown in the FGR-affected placenta [7,13]. It is worth mentioning that *TGIF1* has been shown to participate in the regulation of the expression of several genes, including angiopoietin 2 (*ANGPT2*) and the integrin subunit alpha V (*ITGAV*) involved in angiogenesis [14]. In the case of the *DLX3* gene, its contribution has also been confirmed in the regulation of the expression of the peroxisome proliferator activated receptor gamma (PPARγ) transcription factor, involved in placenta development, trophoblast differentiation, and the occurrence of FGR [3,15].

Considering the genetic changes localized in homeotic genes previously related to FGR, today’s literature indicates single nucleotide polymorphisms (SNPs), both from *DLX3* and *HLX* genes [16,17,18]. For the *DLX3* gene, a certain association was demonstrated between the rs2278163 polymorphism and the occurrence of dental caries in children with higher loads of *Streptococcus mutans* and *Streptococcus sobrinus* [18]. Additionally, a weak correlation was found between the incidence of alleles in *DLX3* rs10459948, localized near rs2278163, and a susceptibility to dental caries in children with higher loads of *Streptococcus mutans* [18]. In the case of rs2278163, some involvement was also demonstrated in the susceptibility to molar–incisor hypomineralization [19]. With regard to the *HLX* gene, several polymorphisms were associated with the clinical course of Graves’ disease (GD), the expression and secretion of type 1/2 T helper (Th1/Th2) cell line cytokines in neonates after birth, the onset of asthma in children, and the development of treatment-dependent acute myeloid leukemia [16,17,20,21]. In the case of *HLX* rs3806325 and rs2184648 polymorphisms, the presence of allelic variants −1407 T and 2742 G, respectively, was significantly associated with a reduced *HLX* promoter transactivation, which was accompanied by an almost complete decrease in the binding of the specificity protein-transcription factors to that region [21]. Regarding the angiogenesis-related genes, the *ANGPT2* −35 G > C polymorphism was recently shown to be significantly correlated with high C-reactive protein levels and severity scores in patients with sepsis [22]. Among the *ITGAV* polymorphisms, both rs3911238 and rs3768777 were associated with rheumatoid arthritis, while rs3768777 was also correlated with a severe progression of primary biliary cirrhosis [23,24,25].

No studies have previously been performed to determine the possible role of SNPs from the *DLX3* and *HLX* homeotic genes, as well as from the angiogenesis-related *ANGPT2* and *ITGAV* genes, in the pathogenesis of FGR. Therefore, we designed a case–control genetic association study to demonstrate the contribution of *DLX3* (rs11656951, rs2278163, rs10459948), *HLX* (rs2184658 and 868058), *ANGPT2* (−35 G > C), and *ITGAV* (rs3911238 and rs3768777) polymorphisms in the occurrence of FGR.

## 2. Materials and Methods

### 2.1. Characteristics of Pregnant Women

A cohort of 380 women with singleton pregnancies included in our study consisted of 190 individuals with FGR and 190 healthy full-term (from 37 to 42 weeks) controls (see Table 1), being inpatients of the Department of Perinatology, Obstetrics and Gynecology, as well as of the Department of Obstetrics and Gynecology of the Polish Mother’s Memorial Hospital-Research Institute (PMMH-RI), in Lodz, Poland. Maternal blood samples were prospectively collected from all the pregnant women on admission during the period from August 2016 to March 2021. Among the diagnosed FGR cases, 58 (30.5%) women were characterized as early-onset FGRs, with the diagnosis obtained between the 18th and the 32nd week of gestation, and 129 (67.9%) were late-onset FGRs, where the diagnosis was obtained from the 32nd week to the 40th week of pregnancy (see Table S1). The women with FGR and early-onset FGR were 15 to 43 years old, while the control group was 19 to 43 years old. The patients with late-onset FGR ranged from 16 to 43 years of age. Early-onset preeclampsia (PE) was diagnosed in 7 (12.1%) women with early-onset FGR. The control group included women after 37 weeks of pregnancy and without FGR, admitted to the department for delivery. The exclusion criteria from the study included: multiple pregnancy, congenital anomalies, genetic syndrome, structural uterine defects, endometriosis, two-vessel umbilical cord, and fetal abnormalities. FGR was diagnosed by ultrasound when EFW was below the 10th percentile in relation to the gestational age and Doppler abnormalities were found, and/or EFW was below the 3rd percentile. The Fetal Medicine Barcelona (FMB) calculator [26] was used to assess percentiles based on ultrasound EFW, determined from biparietal diameter (BPD), head circumference (HC), abdominal circumference (AC), femur length (FL), and humerus length (HL), as well as umbilical artery (UA) and middle cerebral artery (MCA) pulsation indices, UA diastolic flow, and uterine artery (Ut.A) flows, estimated by Doppler ultrasound.

See Table 1 and Table S1 for detailed data on the number of pregnancies and the occurrence of certain pregnancy disorders among the studied pregnant women, including anemia, asthma and respiratory infections, bleeding, diabetes mellitus (DM), hypothyroidism, threatened miscarriage, thrombocytopenia and urogenital infections. The activated partial thromboplastin time (APTT) and platelet (PLT) parameters, including PLT count, platelet distribution width (PDW), mean platelet volume (MPV) and plateletcrit (PCT), are also presented for the women enrolled into the study. EFW was below the 10th percentile in FGR cases, while among the controls, it was between the 11th and the 100th percentiles, estimated by the FMB calculator. The study was approved by the Research Ethics Committee at the PMMH-RI (approval numbers 31/2018 and 13/2019). Informed consent forms were signed by all the invited pregnant women, as recommended by the Research Ethics Committee.

### 2.2. Blood Sample Collection and Analysis

Peripheral venous blood samples were collected on admission from each pregnant woman enrolled into the study, both for diagnostic and research purposes, being anonymized in the latter instance. Nine NC/1.4 mL coagulation tubes were used to evaluate APTT using the HemosIL APTT-SP reagent on an ACL TOP 550 CTS automated system (Instrumentation Laboratory, Werfen Company, Bedford, MA, USA). The APTT reference range was 23 to 36.9 s, according to the manufacturer. EDTA KE/1.2 mL tubes were used for complete blood count (CBC) and DNA extraction. PLT parameters were estimated as a part of the CBC complex using the Fluorocell PLT reagent on a Sysmex XN-2000 Automated Hematology System (Sysmex, Kobe, Japan). The PLT count was referenced between 150 × 10^9^/L and 400 × 10^9^/L, and the MPV was normal from 8.0 to 10.0 fL, as reported by the manufacturer (Sysmex, Kobe, Japan).

Total DNA was purified from 200 μL of whole-blood samples using a Syngen Blood/Cell DNA Mini Kit (Syngen Biotech, Wroclaw, Poland). The obtained DNA was eluted from a mini spin column in 100 μL of DE buffer and stored at −20 °C until further analysis.

### 2.3. PCR-RFLP Assays for ANGPT2, HLX, and ITGAV Polymorphisms

**Figure 1:**
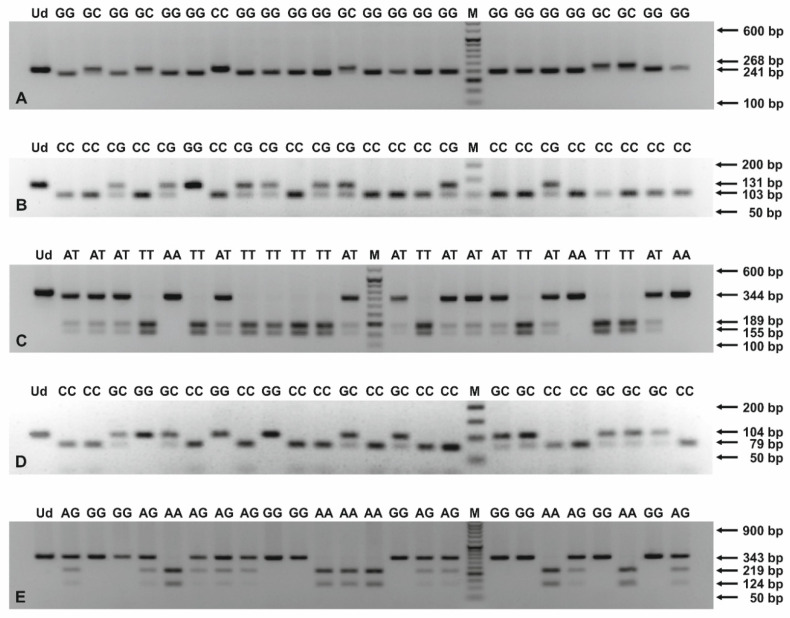
PCR-RFLP products for genotyping of *ANGPT2* −35 G > C (**A**), *HLX* rs2184658 (**B**), rs868058 (**C**), *ITGAV* rs3911238 (**D**), and rs3768777 (**E**) polymorphisms. The PCR products were digested with endonucleases: HindIII (**A**), MnlI (**B**), VspI (**C**), MvaI (**D**), and NlaIII (**E**), and then separated in 2.5–3.4% agarose gels, stained with ethidium bromide. The numbers to the right of the electropherograms show the lengths of separated DNA fragments. M: 50 bp DNA marker; Ud: undigested PCR product; AA, AG, AT, CC, CG, GC, GG, TT: genotypes in studied SNPs.

*ANGPT2* −35 G > C, *HLX* rs2184658 and rs868058, as well as *ITGAV* rs3911238 and rs3768777 polymorphisms, were assayed by PCR-RFLP, as previously described [17,24,27,28,29]. The European minor allele frequencies (MAFs) for SNPs, localized on the *HLX* and *ITGAV* genes, were >10.0%, according to the NCBI Allele Frequency Aggregator (ALFA) project. The primer sequences and PCR-RFLP assay parameters are presented in Table S2. Briefly, PCR mixtures contained up to 0.5 μg of purified DNA, 0.2 mM dNTPs mix, 0.4 μM of each SNP-specific primer, 1 × polymerase B buffer, and 0.5 U of Perpetual Taq DNA Polymerase (EURx, Gdańsk, Poland). The PCR program included initial denaturation at 95 °C for 3 min, 40 cycles of denaturation at 95 °C for 30 s, annealing at 52.7–61.5 °C, depending on polymorphism, for 40 s, an extension at 72 °C for 1 min and a final extension at 72 °C for 7 min. The PCR products were digested with 10 U of the appropriate endonuclease at defined temperatures for 16 h. PCR and restriction digestions were performed on a T100 Thermal Cycler (Bio-Rad, Singapore). The PCR and RFLP products were separated in 1.0–3.4% agarose gels (see Figure 1), prepared in 1 × TAE buffer, depending on the length of analyzed DNA fragments, and visualized in a ChemiDoc XRS+ imaging system (Bio-Rad, Hercules, CA, USA).

### 2.4. Sanger Sequencing for the SNPs Localized on DLX3 Gene

**Figure 2:**
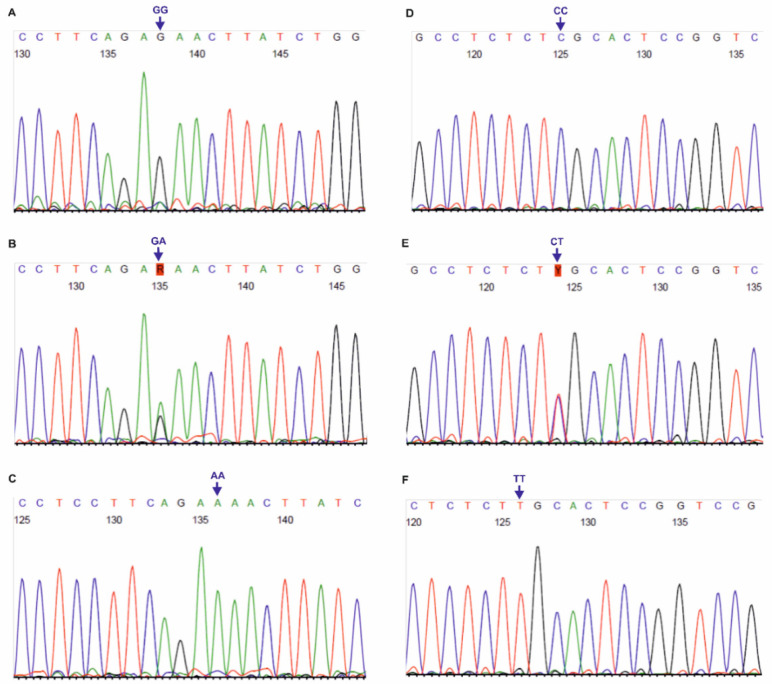
Chromatograms of DNA forward strands for *DLX3* rs11656951 (**A**–**C**) and rs2278163 (**D**–**F**) SNPs. AA, CC, CT, GA, GG, TT: genotypes in the tested polymorphisms.

**Figure 3:**
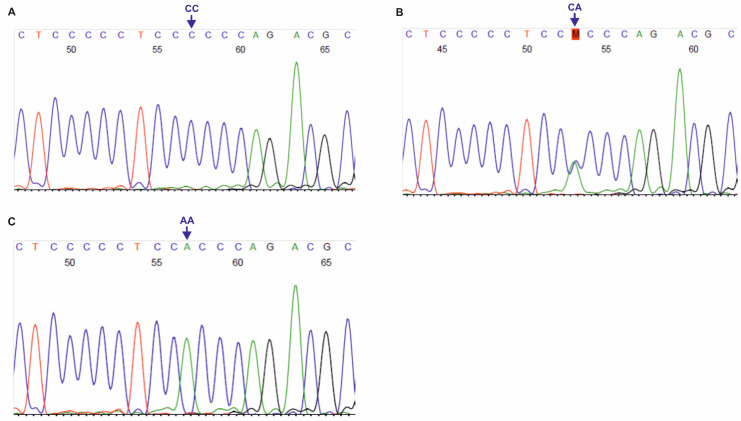
Chromatograms of DNA forward strands for *DLX3* rs10459948 polymorphism (**A**–**C**). AA, CA, CC: genotypes in the tested SNP.

The primer sequences used for genotyping of the three SNPs, localized on *DLX3* gene, i.e., rs11656951, rs2278163 and rs10459948, were self-designed using the PerlPrimer v1.1.21 software, and their specificity was checked by the Primer-BLAST tool [30]. The European MAFs for the tested *DLX3* SNPs were >5.0%, according to the NCBI ALFA project. For rs11656951, PCR products of 331 bp length were obtained, using the following primers For: 5′-CCCACCTTAGACCATCTCTTTCC-3′ and Rev: 5′-CTCTCGCTCCTATGCTCTCC-3′ (primer concentration: 0.4 μM, annealing at 58 °C, 40 cycles). For rs2278163 and rs10459948, 337 bp PCR products were obtained with the primers: For: 5′-CATTTGATTGTGGCTTGGGAC-3′ and Rev: 5′-GTGACAGAAGACTCGGGCAG-3′ (primer concentration: 0.3 μM, annealing at 64 °C, 36 cycles). PCR was performed using Perpetual Taq DNA Polymerase (EURx, Gdańsk, Poland), similarly to the PCR-RFLP assays described in this study. PCR products were verified in 1.0% agarose gels and purified using the ExoSAP-IT™ PCR Product Cleanup Reagent (Thermo Fisher Scientific, Waltham, MA, USA). Sanger sequencing was performed with forward primers, using the BigDye™ Terminator v3.1 Cycle Sequencing Kit (Thermo Fisher Scientific), on a 3500 Genetic Analyzer (Thermo Fisher Scientific). Chromatograms (see Figure 2 and Figure 3) were analyzed using the Sequence Scanner 1.0 software (Applied Biosystems, Waltham, MA, USA).

### 2.5. Statistical Analysis

Descriptive statistics of the pregnant women were performed using NCSS 2004 software. Pearson’s chi-square test was used to determine differences between the studied groups of pregnant women in the number of pregnancies, the occurrence of pregnancy disorders, the methods of delivery, and the fetal sex. In order to compare the women in terms of age, APTT, PLT parameters, gestational age at delivery, as well as birth weight and Apgar scores of their offspring, Student’s t-test or Mann–Whitney U test were performed, depending on the normality level of the examined variables. The Hardy–Weinberg equilibrium and the frequencies of alleles and genotypes in the *ANGPT2*, *DLX3*, *HLX*, and *ITGAV* gene polymorphisms were determined using SNPStats software [31]. Relationships between the genotypes in the tested SNPs and the occurrence of FGR, as well as the models of inheritance, were estimated using logistic regression analyses. The distribution of alleles from the studied polymorphisms between the pregnant groups was determined using Pearson’s chi-square test. The results were considered statistically significant at the significance level of *p* ≤ 0.050.

## 3. Results

### 3.1. Females and Offspring with FGR and Healthy Controls

The pregnant women in the study groups were of a similar age (see Table 1 and Table S1). Among FGR cases, multiparity occurred much more frequently compared to healthy controls (*p* = 0.021). Considering pregnancy disorders, anemia, threatened miscarriage, and thrombocytopenia were significantly more frequent in women with FGR than in healthy subjects (*p* ≤ 0.050). Similarly, anemia was more common in the women with early-onset FGR compared to those with late-onset disease (*p* = 0.001). The APTT and PLT parameters reached similar values in the studied groups. A lower gestational age at delivery was observed among the women with FGR compared to healthy ones, and in the women with early-onset FGR than in those with late-onset FGR (*p* ≤ 0.001). Among the FGR cases, caesarean section was significantly more frequent than vaginal delivery vs. the healthy controls (*p* ≤ 0.050). The fetal sex of the offspring had a similar distribution between the studied groups of women. Neonatal birth weight and Apgar scores at 1 and 5 min were significantly lower in the FGRs than in healthy controls and in the early-onset FGR compared to the late-onset disease (*p* ≤ 0.001).

### 3.2. Hardy–Weinberg Equilibrium

The Hardy–Weinberg (H-W) equilibrium was maintained for the genotypes from *DLX3*, *HLX* and *ITGAV* polymorphisms in all the groups of pregnant women (*p* > 0.050). In *ANGPT2* −35 G > C SNP, H-W was observed in FGR cases (*p* > 0.050), while it was not obtained in healthy controls (*p* = 0.027).

### 3.3. Genetic Alterations from ANGPT2, DLX3, HLX, and ITGAV Polymorphisms

The genotypes, defined in *ANGPT2* −35 G > C, *DLX3* rs11656951, rs2278163, rs10459948, *HLX* rs2184658 and rs868058, as well as in *ITGAV* rs3911238 and rs3768777, were similarly distributed among the women with FGR and the healthy controls (see Table S3). A comparison of the women with early-onset FGR and the healthy subjects showed that the AT heterozygotes in *HLX* rs868058 were significantly associated with an approximately two-fold increase in disease risk in the codominant (OR 2.18 95% CI 1.16–4.09, *p* = 0.045, see Table 2) and overdominant models (OR 2.11 95% CI 1.16–3.83, *p* = 0.014). The relationship was significant in the overdominant model (OR 1.99 95% CI 1.07–3.70, *p* = 0.030) and also after the cases of being large for their gestational age (54/190 (28.4%)) were excluded from the control group. That association was significant in both the codominant (OR 2.21 95% CI 1.08–4.53, *p* = 0.028) and overdominant models (OR 2.42 95% CI 1.21–4.82, *p* = 0.011) after the adjustment imposed by anemia. Similarly, the AT heterozygotes in rs868058 were significantly more frequent in the women with early-onset FGR than in those with the late-onset disease in the overdominant model (OR 2.08 95% CI 1.11–3.89, *p* = 0.022, see Table 3). The results from the study also remained significant in the codominant and/or overdominant models when corrected for pregnancy disorders including anemia, asthma and respiratory infections, bleeding, DM, hypothyroidism, miscarriage risk, thrombocytopenia, and urogenital infections (see Table 4 and Table S4). After the adjustment by anemia, the AT heterozygotes in *HLX* rs868058 were found significantly more often in the women with early-onset FGR, compared to those with late-onset disease, also in the codominant model (OR 2.45 95% CI 1.23–4.90, *p* = 0.034). The alleles, localized in all the analyzed SNPs, had a similar distribution pattern among the studied groups of pregnant women (see Tables S5 and S6).

### 3.4. Study Size Calculation

Based on the allele prevalence rates determined for all the polymorphisms analyzed in our populations of women with FGR and healthy controls, a minimum necessary sample size should have been 184 subjects, with a 95% confidence level and a 5% margin of error. Considering the analyses performed for the women with early-onset FGR and healthy controls, the number of enrolled individuals should have been at least 147, while for comparisons of early-onset FGR and late-onset disease, the minimum sample size should have been 123 women. All those results were obtained with respect to the allele frequencies, found for *ITGAV* rs3768777.

Taking into account the European MAFs, provided for SNPs from the *DLX3*, *HLX*, and *ITGAV* genes, as part of the NCBI ALFA project, we calculated that at least 338 pregnant women should have been included in our study. That minimum sample size was obtained for both *ITGAV* rs3768777 and *HLX* rs868058.

## 4. Discussion

The reported study showed that the AT heterozygotes for *HLX* rs868058 had contributed to an approximately two-fold increase in the risk of early-onset FGR in Caucasian pregnant women. It was previously determined that the homeobox gene *HLX* was mainly expressed in proliferating cytotrophoblastic cells during early placenta development [32]. It was then suggested that decreased HLX levels were necessary for cytotrophoblast differentiation, while altered gene expression could be associated with placental pathologies [32]. In term placental explants and the BeWo trophoblast cell line, lowered HLX expression was correlated with a knockdown of the insulin-like growth factor 2 receptor (IGF2R) involved in the regulation of villous trophoblast survival and apoptosis [33]. Noteworthy was the significantly reduced expression of both *HLX* and *ESX1L*, noted from the 27th week of pregnancy, which could be associated with a declined growth rate of the fetus, observed in the third trimester [34,35]. A decreased expression of *HLX* mRNA and protein was found in the placentas from the pregnancies with idiopathic FGR [11,12]. Similarly, a significantly lowered expression of the *HLX* gene at both mRNA and protein levels was determined in the placenta of FGR twins compared to the normal control cases [36]. *HLX* was suggested to have been involved in abnormal placenta development, found in discordant twin pregnancies [36].

Regarding the rs868058 polymorphism of the *HLX* gene, it was only investigated in relation to the development of asthma in children, but no relationship was found [21]. Our results, as reported here, were the first outcomes to suggest a possible clinical relevance of the presented SNP. Rs868058 is located in the third intron of the gene and is included in the non-coding transcript exon variant HLX-203 (transcript ID: ENST0000054919.2), according to the Ensembl genome browser. Moreover, rs868058 is located in the regulatory region ENSR00000020421, typed as a promoter, which—among others—contributes to trophoblast suppression and stable placenta. Regarding the ENSR00000020421 regulatory function, the T allele in rs868058 is indicated as a regulatory region variant, in line with the Ensembl browser. Based on the results of our study, AT heterozygotes appear to be involved in a deregulation process of the trophoblast function, resulting in placental imbalance and followed by early-onset FGR. For other SNPs localized in *HLX*, the association of some polymorphisms with GD, childhood asthma, and therapy-related acute myeloid leukemia (t-AML) was previously confirmed [17,20,21,37]. The G allele in rs2184658, associated with decreased *HLX* expression was more frequently identified in patients with intractable GD, compared to those with GD in remission [17]. We found no relationship in our study between this SNP and FGR. An association with two tagging SNPs, rs3806325 and rs12141189, representing seven *HLX* polymorphisms, was reported in childhood asthma [21]. In turn, an over three-fold increased risk of t-AML was found in carriers of the CT genotype or at least one polymorphic T allele in the rs2738756 polymorphism [20].

The genetic results of our study remained also significant when corrected by pregnancy disorders, including anemia, asthma and respiratory infections, bleeding, DM, hypothyroidism, threatened miscarriage, thrombocytopenia, and urogenital infections. Previously, maternal anemia was, among other things, correlated with preterm birth (PTB), low birth weight, perinatal mortality, and maternal death [38]. The incidence of gestational iron deficiency anemia was significantly much higher in the women with FGR, as well as in PTB [39]. Similarly, maternal anemia was in our study significantly more common in the FGRs, and especially in those with early-onset disease, compared to the healthy controls. Considering pregnant women with moderate or severe asthma, a higher incidence of spontaneous abortion, fetal structural anomalies, PTB, PE, FGR, oligohydramnios, gestational diabetes, and intrauterine fetal death was recently confirmed [40]. In pregnant women with symptomatic asthma, maternal hypoxia was suggested as a possible mechanism of FGR [41]. In our study, asthma and respiratory infections had a similar distribution pattern among the study groups, although the disorder was more frequent in cases of early-onset FGR compared to healthy controls. Conversely, decidual hemorrhage observed in the first trimester was previously associated with later adverse pregnancy outcomes, including fetal loss, PE, abruption, FGR, and PTB [42]. During the second half of pregnancy, independent risk factors for bleeding were reported, including oligo- and polyhydramnios, FGR, previous abortions, and advanced maternal age [43]. Bleeding was in our study more frequent among FGR cases, when compared to healthy controls, but the difference was statistically insignificant. It was also suggested from the first trimester that adverse pregnancy conditions, possibly involved in fetal nutrient restriction, such as smoking, cocaine use, chronic hypertension, anemia, and chronic DM, lead to symmetrical FGR [44]. However, in our study, DM was similarly distributed between the women with FGR and healthy controls.

Regarding subclinical hypothyroidism (SCH), it has recently been linked to FGR, although no effects on the risk of the disease were found for SCH, thyroid peroxidase antibody and isolated hypothyroxinemia [45]. In Bangladesh, overt hypothyroidism predisposed women to pregnancy-induced hypertension, FGR, and gestational diabetes, compared to the subclinical disease [46]. In line with our outcomes, hypothyroidism had a similar distribution among the groups of enrolled pregnant women, although a slightly higher prevalence was observed for FGR, as compared to control cases. Previously, no differences were observed in the incidence of FGR between the groups of women without bleeding and threatened abortion [47]. In our study, threatened miscarriage was found only in the women with FGR, as cases with that diagnosis were excluded from the healthy control group. Considering the mean platelet count, it was observed that the values decreased during gestation in women with pregnancy-related complications, as well as in healthy subjects, starting from the first trimester [48]. However, gestational thrombocytopenia at delivery was more common in the women with adverse pregnancy outcomes compared to healthy controls [48]. In the current study, gestational thrombocytopenia was also more prevalent among the women with FGR, particularly those with the early-onset disease, compared to the healthy controls. Regarding urogenital infections, an increased risk of FGR was previously determined in cases of vaginal and cervical infections with *Bacteroides*, *Prevotella*, *Porphyromonas* spp., *Mycoplasma hominis*, *Ureaplasma urealyticum*, and *Trichomonas vaginalis* [49]. In another study, a colonization of the maternal genital tract with *Chlamydia trachomatis* and *Candida albicans* was also associated with FGR [50]. Currently, we observed a similar distribution of urogenital infections in the studied groups of pregnant women.

In conclusion, the heterozygous AT genotype in *HLX* rs868058 can be considered a significant risk factor for the development of early-onset FGR, regardless of adverse pregnancy outcomes in women. Although early-onset FGR is now diagnosed without difficulty, it is still an incurable disorder of pregnancy. The only available therapeutic strategy for the management of early-onset FGR is to monitor and terminate pregnancy when the risk associated with fetal immaturity is lower than the risk of intrauterine death. It would be extremely important to understand the signaling pathways involved in the heterozygous AT state in *HLX* rs868058 in order to identify targets for new therapeutic approaches that are still needed to treat early-onset FGR.

## Figures and Tables

**Table 1:** Characteristics of women with fetal growth restriction and healthy controls.

		Controls	FGR ^a^ Cases	*p*-Value ^b^	Early-Onset FGR Cases	*p*-Value
**Number**		190	190		58	
**Age [years]**		28.99 ± 4.81	28.80 ± 5.78	0.729	28.84 ± 5.78	0.849
**No. ^c^ of pregnancy, *n* (%)**	**1**	116 (61.1%)	102 (54.0%)	**0.021**	28 (49.1%)	0.192
	**2**	56 (29.5%)	45 (23.8%)		18 (31.6%)	
	**3**	13 (6.8%)	27 (14.3%)		8 (14.0%)	
	**4**	5 (2.6%)	11 (5.8%)		3 (5.3%)	
	**5**	0 (0.0%)	3 (1.6%)		0 (0.0%)	
	**7**	0 (0.0%)	1 (0.5%)		0 (0.0%)	
**Pregnancy disorders, *n* (%)**	**Anemia**	7 (3.7%)	33 (17.4%)	**≤0.001**	18 (31.0%)	**≤0.001**
	**Asthma and respiratory system infections**	5 (2.6%)	4 (2.1%)	0.736	2 (3.4%)	0.742
	**Bleeding**	3 (1.6%)	5 (2.6%)	0.475	1 (1.7%)	0.939
	**Diabetes mellitus**	27 (14.2%)	20 (10.5%)	0.275	5 (8.6%)	0.266
	**Hypothyroidism**	25 (13.2%)	30 (15.8%)	0.466	10 (17.2%)	0.434
	**Threatened miscarriage**	0 (0.0%)	9 (4.7%)	**0.002**	3 (5.2%)	**0.002**
	**Thrombocytopenia**	2 (1.1%)	9 (4.9%)	**0.031**	5 (8.8%)	**0.003**
	**Urogenital infections**	17 (8.9%)	14 (7.4%)	0.574	5 (8.8%)	0.939
**APTT [s] ^d^**		28.0 (23.6–33.9)	28.2 (22.8–39.4)	0.376	28.3 (23.8–34.6)	0.343
**Platelet parameters**	**No. [×10^9^/L]**	205.5 (129.0–398.0)	209.0 (70.0–368.0)	0.378	211.5 (91.0–314.0)	0.834
	**PDW [fL] ^e^**	13.9 (9.0–23.7)	13.8 (9.4–24.6)	0.745	14.1 (9.7–24.6)	0.614
	**MPV [fL] ^f^**	11.27 ± 0.96	11.39 ± 1.09	0.263	11.50 ± 1.14	0.142
	**PCT [%] ^g^**	0.23 (0.16–0.44)	0.24 (0.08–0.41)	0.103	0.24 (0.11–0.39)	0.381
**Gestational age and delivery mode, *n* (%)**	**Weeks**	39 (37–41)	37 (24–41)	**≤0.001**	32.5 (24.0–40.0)	**≤0.001**
	**Vaginal**	80 (42.1%)	48 (25.3%)	**≤0.001**	15 (25.9%)	**0.026**
	**C-section ^h^**	110 (57.9%)	142 (74.7%)		43 (74.1%)	
**Fetal sex, *n* (%)**	**Female**	106 (55.8%)	102 (53.7%)	0.680	32 (55.2%)	0.934
	**Male**	84 (44.2%)	88 (46.3%)		26 (44.8%)	
**Neonatal data**	**Weight [percentiles]**	77 (11–100)	2 (0–9)	**≤0.001**	0 (0–9)	**≤0.001**
	**Apgar in 1 min**	10 (6–10)	9 (0–10)	**≤0.001**	8 (0–10)	**≤0.001**
	**Apgar in 5 min**	10 (7–10)	9 (0–10)	**≤0.001**	8 (0–10)	**≤0.001**

^a^ FGR, fetal growth restriction; ^b^ *p*-value, statistically significant results are marked in bold; ^c^ No., number; ^d^ APTT (s), activated partial thromboplastin time (second); ^e^ PDW, platelet distribution width; ^f^ MPV, mean platelet volume; ^g^ PCT, plateletcrit; ^h^ C-section, caesarean section. Continuous variables are presented as means ± standard deviations or medians (minimum-maximum) and categorical as numbers (%).

**Table 2:** Relationships among the polymorphisms in *ANGPT2*, *DLX3*, *HLX*, and *ITGAV* genes, and the early-onset fetal growth restriction.

Polymorphism	Genetic Model	Genotype	Genotype Prevalence, *n* (%) ^a^		OR ^b^ (95% CI ^c^)	*p*-Value ^d^	AIC ^e^
			Controls	Cases			
***ANGPT2***	Codominant	G/G	169 (89%)	51 (87.9%)	1.00	0.980	275.7
**−35 G > C**		G/C	18 (9.5%)	6 (10.3%)	1.10 (0.42–2.93)		
		C/C	3 (1.6%)	1 (1.7%)	1.10 (0.11–10.85)		
	Dominant	G/G	169 (89%)	51 (87.9%)	1.00	0.830	273.7
		G/C-C/C	21 (11.1%)	7 (12.1%)	1.10 (0.44–2.75)		
	Recessive	G/G-G/C	187 (98.4%)	57 (98.3%)	1.00	0.940	273.8
		C/C	3 (1.6%)	1 (1.7%)	1.09 (0.11–10.72)		
	Overdominant	G/G-C/C	172 (90.5%)	52 (89.7%)	1.00	0.850	273.7
		G/C	18 (9.5%)	6 (10.3%)	1.10 (0.42–2.92)		
***DLX3***	Codominant	G/G	134 (70.5%)	41 (70.7%)	1.00	0.820	275.4
**rs11656951**		G/A	50 (26.3%)	16 (27.6%)	1.05 (0.54–2.03)		
		A/A	6 (3.2%)	1 (1.7%)	0.54 (0.06–4.66)		
	Dominant	G/G	134 (70.5%)	41 (70.7%)	1.00	0.980	273.8
		G/A-A/A	56 (29.5%)	17 (29.3%)	0.99 (0.52–1.89)		
	Recessive	G/G-G/A	184 (96.8%)	57 (98.3%)	1.00	0.540	273.4
		A/A	6 (3.2%)	1 (1.7%)	0.54 (0.06–4.56)		
	Overdominant	G/G-A/A	140 (73.7%)	42 (72.4%)	1.00	0.850	273.7
		G/A	50 (26.3%)	16 (27.6%)	1.07 (0.55–2.06)		
***DLX3***	Codominant	C/C	126 (66.3%)	39 (67.2%)	1.00	0.930	275.6
**rs2278163**		C/T	56 (29.5%)	16 (27.6%)	0.92 (0.48–1.79)		
		T/T	8 (4.2%)	3 (5.2%)	1.21 (0.31–4.79)		
	Dominant	C/C	126 (66.3%)	39 (67.2%)	1.00	0.900	273.8
		C/T-T/T	64 (33.7%)	19 (32.8%)	0.96 (0.51–1.79)		
	Recessive	C/C-C/T	182 (95.8%)	55 (94.8%)	1.00	0.760	273.7
		T/T	8 (4.2%)	3 (5.2%)	1.24 (0.32–4.84)		
	Overdominant	C/C-T/T	134 (70.5%)	42 (72.4%)	1.00	0.780	273.7
		C/T	56 (29.5%)	16 (27.6%)	0.91 (0.47–1.75)		
***DLX3***	Codominant	C/C	168 (88.4%)	52 (89.7%)	1.00	0.760	275.2
**rs10459948**		C/A	21 (11.1%)	6 (10.3%)	0.92 (0.35–2.41)		
		A/A	1 (0.5%)	0 (0%)	0.00 (0.00-NA ^f^)		
	Dominant	C/C	168 (88.4%)	52 (89.7%)	1.00	0.790	273.7
		C/A-A/A	22 (11.6%)	6 (10.3%)	0.88 (0.34–2.29)		
	Recessive	C/C-C/A	189 (99.5%)	58 (100%)	1.00	0.460	273.2
		A/A	1 (0.5%)	0 (0%)	0.00 (0.00-NA)		
	Overdominant	C/C-A/A	169 (89%)	52 (89.7%)	1.00	0.880	273.8
		C/A	21 (11.1%)	6 (10.3%)	0.93 (0.36–2.42)		
***HLX***	Codominant	C/C	109 (57.4%)	40 (69%)	1.00	0.250	273.0
**rs2184658**		C/G	71 (37.4%)	15 (25.9%)	0.58 (0.30–1.12)		
		G/G	10 (5.3%)	3 (5.2%)	0.82 (0.21–3.12)		
	Dominant	C/C	109 (57.4%)	40 (69%)	1.00	0.110	271.2
		C/G-G/G	81 (42.6%)	18 (31%)	0.61 (0.32–1.13)		
	Recessive	C/C-C/G	180 (94.7%)	55 (94.8%)	1.00	0.980	273.8
		G/G	10 (5.3%)	3 (5.2%)	0.98 (0.26–3.69)		
	Overdominant	C/C-G/G	119 (62.6%)	43 (74.1%)	1.00	0.100	271.1
		C/G	71 (37.4%)	15 (25.9%)	0.58 (0.30–1.13)		
***HLX***	Codominant	T/T	100 (52.6%)	21 (36.2%)	1.00	**0.045**	269.6
**rs868058**		A/T	70 (36.8%)	32 (55.2%)	2.18 (1.16–4.09)		
		A/A	20 (10.5%)	5 (8.6%)	1.19 (0.40–3.53)		
	Dominant	T/T	100 (52.6%)	21 (36.2%)	1.00	**0.028**	268.9
		A/T-A/A	90 (47.4%)	37 (63.8%)	1.96 (1.07–3.59)		
	Recessive	T/T-A/T	170 (89.5%)	53 (91.4%)	1.00	0.670	273.6
		A/A	20 (10.5%)	5 (8.6%)	0.80 (0.29–2.24)		
	Overdominant	T/T-A/A	120 (63.2%)	26 (44.8%)	1.00	**0.014**	267.7
		A/T	70 (36.8%)	32 (55.2%)	2.11 (1.16–3.83)		
***ITGAV***	Codominant	C/C	97 (51%)	32 (55.2%)	1.00	0.220	272.8
**rs3911238**		G/C	81 (42.6%)	19 (32.8%)	0.71 (0.38–1.35)		
		G/G	12 (6.3%)	7 (12.1%)	1.77 (0.64–4.88)		
	Dominant	C/C	97 (51%)	32 (55.2%)	1.00	0.580	273.5
		G/C-G/G	93 (49%)	26 (44.8%)	0.85 (0.47–1.53)		
	Recessive	C/C-G/C	178 (93.7%)	51 (87.9%)	1.00	0.170	271.9
		G/G	12 (6.3%)	7 (12.1%)	2.04 (0.76–5.44)		
	Overdominant	C/C-G/G	109 (57.4%)	39 (67.2%)	1.00	0.180	271.9
		G/C	81 (42.6%)	19 (32.8%)	0.66 (0.35–1.22)		
***ITGAV***	Codominant	G/G	75 (39.5%)	26 (44.8%)	1.00	0.420	274.1
**rs3768777**		A/G	90 (47.4%)	22 (37.9%)	0.71 (0.37–1.34)		
		A/A	25 (13.2%)	10 (17.2%)	1.15 (0.49–2.72)		
	Dominant	G/G	75 (39.5%)	26 (44.8%)	1.00	0.470	273.3
		A/G-A/A	115 (60.5%)	32 (55.2%)	0.80 (0.44–1.45)		
	Recessive	G/G-A/G	165 (86.8%)	48 (82.8%)	1.00	0.440	273.2
		A/A	25 (13.2%)	10 (17.2%)	1.38 (0.62–3.06)		
	Overdominant	G/G-A/A	100 (52.6%)	36 (62.1%)	1.00	0.200	272.2
		A/G	90 (47.4%)	22 (37.9%)	0.68 (0.37–1.24)		

^a^ *n*, number; ^b^ OR, odds ratio; ^c^ 95% CI, confidence interval; ^d^ *p*-value, statistically significant results are marked in bold; ^e^ AIC, Akaike information criterion; ^f^ NA, not analyzed. Categorical variables are presented as numbers (%).

**Table 3:** Distribution of genotypes from *ANGPT2*, *DLX3*, *HLX*, and *ITGAV* polymorphisms between the women with early and late-onset FGR.

Polymorphism	Genetic Model	Genotype	Genotype Prevalence, *n* (%) ^a^		OR ^c^ (95% CI ^d^)	*p*-Value ^e^	AIC ^f^
			Late-Onset FGR ^b^	Early-Onset FGR			
***ANGPT2***	Codominant	G/G	114 (88.4%)	51 (87.9%)	1.00	0.850	237.3
**−35 G > C**		G/C	14 (10.8%)	6 (10.3%)	0.96 (0.35–2.63)		
		C/C	1 (0.8%)	1 (1.7%)	2.24 (0.14–36.45)		
	Dominant	G/G	114 (88.4%)	51 (87.9%)	1.00	0.930	235.6
		G/C-C/C	15 (11.6%)	7 (12.1%)	1.04 (0.40–2.71)		
	Recessive	G/G-G/C	128 (99.2%)	57 (98.3%)	1.00	0.570	235.3
		C/C	1 (0.8%)	1 (1.7%)	2.25 (0.14–36.54)		
	Overdominant	G/G-C/C	115 (89.2%)	52 (89.7%)	1.00	0.920	235.6
		G/C	14 (10.8%)	6 (10.3%)	0.95 (0.34–2.60)		
***DLX3***	Codominant	G/G	92 (71.3%)	41 (70.7%)	1.00	0.950	237.5
**rs11656951**		G/A	34 (26.4%)	16 (27.6%)	1.06 (0.52–2.12)		
		A/A	3 (2.3%)	1 (1.7%)	0.75 (0.08–7.41)		
	Dominant	G/G	92 (71.3%)	41 (70.7%)	1.00	0.930	235.6
		G/A-A/A	37 (28.7%)	17 (29.3%)	1.03 (0.52–2.04)		
	Recessive	G/G-G/A	126 (97.7%)	57 (98.3%)	1.00	0.790	235.5
		A/A	3 (2.3%)	1 (1.7%)	0.74 (0.08–7.24)		
	Overdominant	G/G-A/A	95 (73.6%)	42 (72.4%)	1.00	0.860	235.6
		G/A	34 (26.4%)	16 (27.6%)	1.06 (0.53–2.14)		
***DLX3***	Codominant	C/C	90 (69.8%)	39 (67.2%)	1.00	0.790	237.1
**rs2278163**		C/T	35 (27.1%)	16 (27.6%)	1.05 (0.52–2.13)		
		T/T	4 (3.1%)	3 (5.2%)	1.73 (0.37–8.10)		
	Dominant	C/C	90 (69.8%)	39 (67.2%)	1.00	0.730	235.5
		C/T-T/T	39 (30.2%)	19 (32.8%)	1.12 (0.58–2.19)		
	Recessive	C/C-C/T	125 (96.9%)	55 (94.8%)	1.00	0.500	235.1
		T/T	4 (3.1%)	3 (5.2%)	1.70 (0.37–7.87)		
	Overdominant	C/C-T/T	94 (72.9%)	42 (72.4%)	1.00	0.950	235.6
		C/T	35 (27.1%)	16 (27.6%)	1.02 (0.51–2.05)		
***DLX3***	---	C/C	117 (90.7%)	52 (89.7%)	1.00	0.820	235.5
**rs10459948**		C/A	12 (9.3%)	6 (10.3%)	1.12 (0.40–3.16)		
***HLX***	Codominant	C/C	76 (58.9%)	40 (69%)	1.00	0.390	235.7
**rs2184658**		C/G	46 (35.7%)	15 (25.9%)	0.62 (0.31–1.24)		
		G/G	7 (5.4%)	3 (5.2%)	0.81 (0.20–3.32)		
	Dominant	C/C	76 (58.9%)	40 (69%)	1.00	0.190	233.8
		C/G-G/G	53 (41.1%)	18 (31%)	0.65 (0.33–1.25)		
	Recessive	C/C-C/G	122 (94.6%)	55 (94.8%)	1.00	0.940	235.6
		G/G	7 (5.4%)	3 (5.2%)	0.95 (0.24–3.81)		
	Overdominant	C/C-G/G	83 (64.3%)	43 (74.1%)	1.00	0.180	233.8
		C/G	46 (35.7%)	15 (25.9%)	0.63 (0.32–1.25)		
***HLX***	Codominant	T/T	69 (53.5%)	21 (36.2%)	1.00	0.063	232.1
**rs868058**		A/T	48 (37.2%)	32 (55.2%)	2.19 (1.13–4.25)		
		A/A	12 (9.3%)	5 (8.6%)	1.37 (0.43–4.33)		
	Dominant	T/T	69 (53.5%)	21 (36.2%)	1.00	**0.028**	230.8
		A/T-A/A	60 (46.5%)	37 (63.8%)	2.03 (1.07–3.83)		
	Recessive	T/T-A/T	117 (90.7%)	53 (91.4%)	1.00	0.880	235.6
		A/A	12 (9.3%)	5 (8.6%)	0.92 (0.31–2.74)		
	Overdominant	T/T-A/A	81 (62.8%)	26 (44.8%)	1.00	**0.022**	230.3
		A/T	48 (37.2%)	32 (55.2%)	2.08 (1.11–3.89)		
***ITGAV***	Codominant	C/C	57 (44.2%)	32 (55.2%)	1.00	0.210	234.4
**rs3911238**		G/C	60 (46.5%)	19 (32.8%)	0.56 (0.29–1.11)		
		G/G	12 (9.3%)	7 (12.1%)	1.04 (0.37–2.90)		
	Dominant	C/C	57 (44.2%)	32 (55.2%)	1.00	0.160	233.7
		G/C-G/G	72 (55.8%)	26 (44.8%)	0.64 (0.34–1.20)		
	Recessive	C/C-G/C	117 (90.7%)	51 (87.9%)	1.00	0.570	235.3
		G/G	12 (9.3%)	7 (12.1%)	1.34 (0.50–3.60)		
	Overdominant	C/C-G/G	69 (53.5%)	39 (67.2%)	1.00	0.076	232.4
		G/C	60 (46.5%)	19 (32.8%)	0.56 (0.29–1.07)		
***ITGAV***	Codominant	G/G	54 (41.9%)	26 (44.8%)	1.00	0.350	235.5
**rs3768777**		A/G	61 (47.3%)	22 (37.9%)	0.75 (0.38–1.47)		
		A/A	14 (10.8%)	10 (17.2%)	1.48 (0.58–3.79)		
	Dominant	G/G	54 (41.9%)	26 (44.8%)	1.00	0.700	235.4
		A/G-A/A	75 (58.1%)	32 (55.2%)	0.89 (0.47–1.65)		
	Recessive	G/G-A/G	115 (89.2%)	48 (82.8%)	1.00	0.240	234.2
		A/A	14 (10.8%)	10 (17.2%)	1.71 (0.71–4.12)		
	Overdominant	G/G-A/A	68 (52.7%)	36 (62.1%)	1.00	0.230	234.2
		A/G	61 (47.3%)	22 (37.9%)	0.68 (0.36–1.28)		

^a^ *n*, number; ^b^ FGR, fetal growth restriction; ^c^ OR, odds ratio; ^d^ 95% CI, confidence interval; ^e^ *p*-value, statistically significant results are marked in bold; ^f^ AIC, Akaike information criterion. Categorical variables are presented as numbers (%).

**Table 4:** Association of *HLX* rs868058 genotypes with early-onset fetal growth restriction, corrected for adverse pregnancy outcomes.

Pregnancy Disorders	Genetic Model	Genotype	Genotype Prevalence, *n* (%) ^a^		OR ^b^ (95% CI ^c^)	*p*-Value ^d^	AIC ^e^
			Controls	Cases			
**Anemia**	Codominant	T/T	100 (52.6%)	21 (36.2%)	1.00	**0.034**	240.7
		A/T	70 (36.8%)	32 (55.2%)	2.19 (1.11–4.33)		
		A/A	20 (10.5%)	5 (8.6%)	0.73 (0.21–2.58)		
	Dominant	T/T	100 (52.6%)	21 (36.2%)	1.00	0.068	242.1
		A/T-A/A	90 (47.4%)	37 (63.8%)	1.82 (0.95–3.50)		
	Recessive	T/T-A/T	170 (89.5%)	53 (91.4%)	1.00	0.220	244.0
		A/A	20 (10.5%)	5 (8.6%)	0.49 (0.15–1.64)		
	Overdominant	T/T-A/A	120 (63.2%)	26 (44.8%)	1.00	**0.011**	239.0
		A/T	70 (36.8%)	32 (55.2%)	2.31 (1.21–4.44)		
**Asthma and respiratory system infections**	Codominant	T/T	100 (52.6%)	21 (36.2%)	1.00	**0.046**	271.5
		A/T	70 (36.8%)	32 (55.2%)	2.17 (1.16–4.08)		
		A/A	20 (10.5%)	5 (8.6%)	1.19 (0.40–3.52)		
	Dominant	T/T	100 (52.6%)	21 (36.2%)	1.00	**0.028**	270.8
		A/T-A/A	90 (47.4%)	37 (63.8%)	1.95 (1.07–3.59)		
	Recessive	T/T-A/T	170 (89.5%)	53 (91.4%)	1.00	0.660	275.5
		A/A	20 (10.5%)	5 (8.6%)	0.80 (0.29–2.23)		
	Overdominant	T/T-A/A	120 (63.2%)	26 (44.8%)	1.00	**0.014**	269.6
		A/T	70 (36.8%)	32 (55.2%)	2.11 (1.16–3.83)		
**Bleeding**	Codominant	T/T	100 (52.6%)	21 (36.2%)	1.00	**0.045**	271.6
		A/T	70 (36.8%)	32 (55.2%)	2.19 (1.16–4.11)		
		A/A	20 (10.5%)	5 (8.6%)	1.20 (0.40–3.56)		
	Dominant	T/T	100 (52.6%)	21 (36.2%)	1.00	**0.027**	270.9
		A/T-A/A	90 (47.4%)	37 (63.8%)	1.97 (1.07–3.62)		
	Recessive	T/T-A/T	170 (89.5%)	53 (91.4%)	1.00	0.670	275.6
		A/A	20 (10.5%)	5 (8.6%)	0.80 (0.29–2.24)		
	Overdominant	T/T-A/A	120 (63.2%)	26 (44.8%)	1.00	**0.013**	269.7
		A/T	70 (36.8%)	32 (55.2%)	2.12 (1.17–3.84)		
**Diabetes mellitus**	Codominant	T/T	100 (52.6%)	21 (36.2%)	1.00	0.052	270.5
		A/T	70 (36.8%)	32 (55.2%)	2.14 (1.14–4.02)		
		A/A	20 (10.5%)	5 (8.6%)	1.15 (0.39–3.42)		
	Dominant	T/T	100 (52.6%)	21 (36.2%)	1.00	**0.034**	269.9
		A/T-A/A	90 (47.4%)	37 (63.8%)	1.92 (1.04–3.52)		
	Recessive	T/T-A/T	170 (89.5%)	53 (91.4%)	1.00	0.630	274.2
		A/A	20 (10.5%)	5 (8.6%)	0.78 (0.28–2.18)		
	Overdominant	T/T-A/A	120 (63.2%)	26 (44.8%)	1.00	**0.016**	268.6
		A/T	70 (36.8%)	32 (55.2%)	2.08 (1.15–3.78)		
**Hypothyroidism**	Codominant	T/T	100 (52.6%)	21 (36.2%)	1.00	**0.050**	271.2
		A/T	70 (36.8%)	32 (55.2%)	2.16 (1.15–4.06)		
		A/A	20 (10.5%)	5 (8.6%)	1.21 (0.41–3.59)		
	Dominant	T/T	100 (52.6%)	21 (36.2%)	1.00	**0.029**	270.4
		A/T-A/A	90 (47.4%)	37 (63.8%)	1.95 (1.06–3.58)		
	Recessive	T/T-A/T	170 (89.5%)	53 (91.4%)	1.00	0.700	275.0
		A/A	20 (10.5%)	5 (8.6%)	0.82 (0.29–2.30)		
	Overdominant	T/T-A/A	120 (63.2%)	26 (44.8%)	1.00	**0.015**	269.3
		A/T	70 (36.8%)	32 (55.2%)	2.09 (1.15–3.79)		
**Threatened miscarriage**	Codominant	T/T	100 (52.6%)	21 (36.2%)	1.00	0.060	263.3
		A/T	70 (36.8%)	32 (55.2%)	2.14 (1.13–4.08)		
		A/A	20 (10.5%)	5 (8.6%)	1.25 (0.42–3.72)		
	Dominant	T/T	100 (52.6%)	21 (36.2%)	1.00	**0.033**	262.4
		A/T-A/A	90 (47.4%)	37 (63.8%)	1.94 (1.05–3.61)		
	Recessive	T/T-A/T	170 (89.5%)	53 (91.4%)	1.00	0.750	266.8
		A/A	20 (10.5%)	5 (8.6%)	0.85 (0.30–2.38)		
	Overdominant	T/T-A/A	120 (63.2%)	26 (44.8%)	1.00	**0.020**	261.5
		A/T	70 (36.8%)	32 (55.2%)	2.06 (1.12–3.78)		
**Thrombocytopenia**	Codominant	T/T	96 (52.2%)	20 (35.1%)	1.00	0.052	258.4
		A/T	69 (37.5%)	32 (56.1%)	2.22 (1.15–4.25)		
		A/A	19 (10.3%)	5 (8.8%)	1.38 (0.46–4.15)		
	Dominant	T/T	96 (52.2%)	20 (35.1%)	1.00	**0.024**	257.2
		A/T-A/A	88 (47.8%)	37 (64.9%)	2.04 (1.09–3.82)		
	Recessive	T/T-A/T	165 (89.7%)	52 (91.2%)	1.00	0.860	262.2
		A/A	19 (10.3%)	5 (8.8%)	0.91 (0.32–2.57)		
	Overdominant	T/T-A/A	115 (62.5%)	25 (43.9%)	1.00	**0.018**	256.7
		A/T	69 (37.5%)	32 (56.1%)	2.09 (1.13–3.86)		
**Urogenital infections**	Codominant	T/T	100 (52.6%)	21 (36.2%)	1.00	**0.045**	271.6
		A/T	70 (36.8%)	32 (55.2%)	2.18 (1.16–4.09)		
		A/A	20 (10.5%)	5 (8.6%)	1.19 (0.40–3.54)		
	Dominant	T/T	100 (52.6%)	21 (36.2%)	1.00	**0.028**	270.9
		A/T-A/A	90 (47.4%)	37 (63.8%)	1.96 (1.07–3.59)		
	Recessive	T/T-A/T	170 (89.5%)	53 (91.4%)	1.00	0.670	275.6
		A/A	20 (10.5%)	5 (8.6%)	0.80 (0.29–2.24)		
	Overdominant	T/T-A/A	120 (63.2%)	26 (44.8%)	1.00	**0.014**	269.7
		A/T	70 (36.8%)	32 (55.2%)	2.11 (1.16–3.83)		

^a^ *n*, number; ^b^ OR, odds ratio; ^c^ 95% CI, confidence interval; ^d^ *p*-value, statistically significant results are marked in bold; ^e^ AIC, Akaike information criterion. Categorical variables are presented as numbers (%).

## Data Availability

All data and materials as well as software application support the published claims and comply with field standards.

## Supplementary Materials

The following supporting information can be downloaded at: https://www.mdpi.com/article/10.3390/biology11030447/s1. Table S1: Characteristics of women with early and late-onset fetal growth restriction. Table S2: Selected PCR-RFLP parameters for genotyping of single nucleotide polymorphisms, localized in *HLX*, *ITGAV*, and *ANGPT2* genes. Table S3: Distribution of the genotypes from *ANGPT2*, *DLX3*, *HLX*, and *ITGAV* polymorphisms between women with FGR and healthy controls. Table S4: Distribution of *HLX* rs868058 genotypes between women with early-onset and late-onset FGR, adjusted by adverse pregnancy outcomes. Table S5: Distribution of the alleles from the *ANGPT2*, *DLX3*, *HLX*, and *ITGAV* SNPs between women with fetal growth restriction and healthy controls. Table S6: Distribution of the alleles from the *ANGPT2*, *DLX3*, *HLX*, and *ITGAV* polymorphisms between women with early and late-onset FGR.
