# Supplementary material for: Rs868058 in the Homeobox Gene HLX Contributes to Early-Onset Fetal Growth Restriction
Source: Biology (Basel). 2022 Mar 16;11(3):447. doi: 10.3390/biology11030447 (PMC8945724; doi:10.3390/biology11030447)
Supplement: Supplementary file 1 [file biology-11-00447-s001.zip › biology-1594996-supplementary.pdf]

biology

MDPI

Article

Rs868058 in the Homeobox Gene *HLX* Contributes to Early-Onset Fetal Growth Restriction

Wioletta Izabela Wujcicka [1,*], Marian Kacerovsky [2,3], Michał Krekora [4,5], Piotr Kaczmarek [6], Beata Leśniczak [7] and Mariusz Grzesiak [5,8]

[1] Scientific Laboratory of the Center of Medical Laboratory Diagnostics and Screening, Polish Mother's Memorial Hospital-Research Institute, 93-338 Lodz, Poland
[2] Department of Obstetrics and Gynecology, University Hospital Hradec Kralove, Charles University, 500 03 Hradec Kralove, Czech Republic; marian.kacerovsky@gmail.com
[3] Biomedical Research Center, University Hospital Hradec Kralove, 500 03 Hradec Kralove, Czech Republic
[4] Department of Obstetrics and Gynecology, Polish Mother's Memorial Hospital-Research Institute, 93-338 Lodz, Poland; krekoram@poczta.onet.pl
[5] Department of Gynecology and Obstetrics, Medical University of Lodz, 93-338 Lodz, Poland; mariusz.grzesiak@gmail.com
[6] Laboratory of Prenatal Fetal and Maternal Diagnostics, Polish Mother's Memorial Hospital-Research Institute, 93-338 Lodz, Poland; kaczmarekpiotr1@gmail.com
[7] 2nd Department of Gynecology and Obstetrics, Medical University of Lodz, 90-251 Lodz, Poland; beata.lesniczak@umed.lodz.pl
[8] Department of Perinatology, Obstetrics and Gynecology, Polish Mother's Memorial Hospital-Research Institute, 93-338 Lodz, Poland
* Correspondence: wioletta.wujcicka@iczmp.edu.pl or wwujcicka@yahoo.com; Tel.: +48-422711520; Fax: +48-422711510

Table S1. Characteristics of women with early and late-onset fetal growth restriction.

		Late-onset FGR [a] cases	Early-onset FGR cases	*P*-value [b]
**Number**		129	58	
**Age [years]**		28.97 ± 5.72	28.84 ± 5.78	0.891
**No. [c] of pregnancy, n (%)**	**1**	71 (55.04%)	28 (49.12%)	0.556
	**2**	27 (20.93%)	18 (31.58%)	
	**3**	19 (14.73%)	8 (14.04%)	
	**4**	8 (6.20%)	3 (5.26%)	
	**5**	3 (2.33%)	0 (0.00%)	
	**7**	1 (0.77%)	0 (0.00%)	
**Pregnancy disorders, n (%)**	**Anemia**	15 (11.6%)	18 (31.0%)	**0.001**
	**Asthma and respiratory system infections**	2 (1.6%)	2 (3.4%)	0.407
	**Bleeding**	4 (3.1%)	1 (1.7%)	0.589
	**Diabetes mellitus**	15 (11.6%)	5 (8.6%)	0.538
	**Hypothyroidism**	19 (14.7%)	10 (17.2%)	0.661
	**Threatened miscarriage**	6 (4.7%)	3 (5.2%)	0.878
	**Thrombocytopenia**	4 (3.2%)	5 (8.8%)	0.108
	**Urogenital infections**	9 (7.0%)	5 (8.8%)	0.693
**APTT [s] [d]**		28.2 (22.8-39.4)	28.3 (23.8-34.6)	0.672
**Platelet parameters**	**No. [x10^9/L]**	218.3 ± 55.6	211.8 ± 56.7	0.471
	**PDW [fL] [e]**	13.7 (9.4-23.3)	14.1 (9.7-24.6)	0.730
	**MPV [fL] [f]**	11.34 ± 1.07	11.50 ± 1.14	0.386
	**PCT [%] [g]**	0.24 ± 0.05	0.24 ± 0.06	0.679
**Gestational age and delivery mode, n (%)**	**Weeks**	37.0 (32.0-40.0)	32.5 (24.0-40.0)	**≤ 0.001**
	**Vaginal**	31 (24.0%)	15 (25.9%)	0.788
	**C-section [h]**	98 (76.0%)	43 (74.1%)	
**Fetal sex, n (%)**	**Female**	69 (53.5%)	32 (55.2%)	0.831
	**Male**	60 (46.5%)	26 (44.8%)	
**Neonatal data**	**Weight [percentiles]**	2 (0-9)	0 (0-9)	**≤ 0.001**
	**Apgar in 1 min**	9 (1-10)	8 (0-10)	**≤ 0.001**
	**Apgar in 5 min**	10 (4-10)	8 (0-10)	**≤ 0.001**

[a] FGR, fetal growth restriction; [b] *P*-value, statistically significant results are marked in bold; [c] No., number; [d] APTT (s), activated partial thromboplastin time (second); [e] PDW, platelet distribution width; [f] MPV, mean platelet volume; [g] PCT, plateletcrit; [h] C-section, caesarean section.

Continuous variables are presented as means ± standard deviations or medians (minimum-maximum) and categorical as numbers (%).

Table S2. Selected PCR-RFLP parameters for genotyping of single nucleotide polymorphisms, localized in *HLX*, *ITGAV*, and *ANGPT2* genes.

Gene	SNP [a]	Alleles	MAF [b]	Primer sequences (5'-3')	Annealing temperature [°C]	Restriction enzyme	Genotypes [bp] [c]	Agarose gel [%]
HLX	**rs2184658**	C>G	12.6	F: GCAGAGCCCTAAAGAGTGAG	52.7	MnlI	CC: 103, 28	3.0
				R: CTAACTTCTGCAGCTCCGAA			CG: 131, 103, 28	
							GG: 131	
HLX	**rs868058**	A>T	34.2	F: GTCTGCTTGGAGTGGCCACACATTAATAGG	61.5	VspI	AA: 344, 23	2.5
				R: CAGCAATTTGGGGACCAAGGAGC			AT: 344, 189, 155, 23	
							TT: 189, 155, 23	
ITGAV	**rs3911238**	G>C	27.2	F: TGGGCACCAGACAATGTTTA	57.0	MvaI	GG: 104	3.4
				R: AGTGAATGTCTCTTTCCCTCAA			GC: 104, 79, 25	
							CC: 79, 25	
ITGAV	**rs3768777**	A>G	32.7	F: AAGTTGCCAACGTTCCGCGTTGCA	58.0	NlaIII	AA: 219, 124	2.5
				R: GTAGTAGAAGATGGTCCTATCCACG			AG: 343, 219, 124	
							GG: 343	
ANGPT2	**-35 G>C**	G>C	-	F: GACCGTGAAAGCTGCTCTGTAAAAGC	58.0	HindIII	GG: 241, 27	3.0
				R: TCAGTAATAAACCAGCAGCTGAGCAAG			GC: 268, 241, 27	
							CC: 268	

[a] SNP, single nucleotide polymorphism; [b] MAF, minor allele frequency; [c] bp, base pair.

Table S3. Distribution of the genotypes from *ANGPT2*, *DLX3*, *HLX*, and *ITGAV* polymorphisms between women with FGR and healthy controls.

Polymorphism	Genetic model	Genotype	Genotype prevalence, n (%) [a]		OR [b] (95 % CI [c])	*P*-value [d]	AIC [e]
			Controls	Cases			
ANGPT2	Codominant	G/G	169 (89%)	167 (87.9%)	1.00		
**-35 G>C**		G/C	18 (9.5%)	21 (11.1%)	1.18 (0.61-2.30)	0.800	532.3
		C/C	3 (1.6%)	2 (1.1%)	0.67 (0.11-4.09)		
	Dominant	G/G	169 (89%)	167 (87.9%)	1.00	0.750	530.7
		G/C-C/C	21 (11.1%)	23 (12.1%)	1.11 (0.59-2.08)		
	Recessive	G/G-G/C	187 (98.4%)	188 (99%)	1.00	0.650	530.6
		C/C	3 (1.6%)	2 (1.1%)	0.66 (0.11-4.01)		
	Overdominant	G/G-C/C	172 (90.5%)	169 (89%)	1.00	0.610	530.5
		G/C	18 (9.5%)	21 (11.1%)	1.19 (0.61-2.31)		
DLX3	Codominant	G/G	134 (70.5%)	134 (70.5%)	1.00		
**rs11656951**		G/A	50 (26.3%)	52 (27.4%)	1.04 (0.66-1.64)	0.800	532.3
		A/A	6 (3.2%)	4 (2.1%)	0.67 (0.18-2.42)		
	Dominant	G/G	134 (70.5%)	134 (70.5%)	1.00	1.000	530.8
		G/A-A/A	56 (29.5%)	56 (29.5%)	1.00 (0.64-1.55)		
	Recessive	G/G-G/A	184 (96.8%)	186 (97.9%)	1.00	0.520	530.4
		A/A	6 (3.2%)	4 (2.1%)	0.66 (0.18-2.38)		
	Overdominant	G/G-A/A	140 (73.7%)	138 (72.6%)	1.00	0.820	530.7
		G/A	50 (26.3%)	52 (27.4%)	1.06 (0.67-1.66)		
DLX3	Codominant	C/C	126 (66.3%)	129 (67.9%)	1.00		
**rs2278163**		C/T	56 (29.5%)	54 (28.4%)	0.94 (0.60-1.47)	0.930	532.7
		T/T	8 (4.2%)	7 (3.7%)	0.85 (0.30-2.43)		
	Dominant	C/C	126 (66.3%)	129 (67.9%)	1.00	0.740	530.7
		C/T-T/T	64 (33.7%)	61 (32.1%)	0.93 (0.61-1.43)		
	Recessive	C/C-C/T	182 (95.8%)	183 (96.3%)	1.00	0.790	530.7

		T/T	8 (4.2%)	7 (3.7%)	0.87 (0.31-2.45)		
	Overdominant	C/C-T/T	134 (70.5%)	136 (71.6%)	1.00	0.820	530.7
		C/T	56 (29.5%)	54 (28.4%)	0.95 (0.61-1.48)		
DLX3 **rs10459948**	Codominant	C/C	168 (88.4%)	171 (90%)	1.00		
		C/A	21 (11.1%)	19 (10%)	0.89 (0.46-1.71)	0.470	531.3
		A/A	1 (0.5%)	0 (0%)	0.00 (0.00-NA [f])		
	Dominant	C/C	168 (88.4%)	171 (90%)	1.00		
		C/A-A/A	22 (11.6%)	19 (10%)	0.85 (0.44-1.62)	0.620	530.5
	Recessive	C/C-C/A	189 (99.5%)	190 (100%)	1.00		
		A/A	1 (0.5%)	0 (0%)	0.00 (0.00-NA)	0.240	529.4
	Overdominant	C/C-A/A	169 (89%)	171 (90%)	1.00	0.740	530.7
		C/A	21 (11.1%)	19 (10%)	0.89 (0.46-1.72)		
HLX **rs2184658**	Codominant	C/C	109 (57.4%)	119 (62.6%)	1.00		
		C/G	71 (37.4%)	61 (32.1%)	0.79 (0.51-1.21)	0.550	531.6
		G/G	10 (5.3%)	10 (5.3%)	0.92 (0.37-2.29)		
	Dominant	C/C	109 (57.4%)	119 (62.6%)	1.00		
		C/G-G/G	81 (42.6%)	71 (37.4%)	0.80 (0.53-1.21)	0.290	529.7
	Recessive	C/C-C/G	180 (94.7%)	180 (94.7%)	1.00		
		G/G	10 (5.3%)	10 (5.3%)	1.00 (0.41-2.46)	1.000	530.8
	Overdominant	C/C-G/G	119 (62.6%)	129 (67.9%)	1.00	0.280	529.6
		C/G	71 (37.4%)	61 (32.1%)	0.79 (0.52-1.21)		
HLX **rs868058**	Codominant	T/T	100 (52.6%)	90 (47.4%)	1.00		
		A/T	70 (36.8%)	82 (43.2%)	1.30 (0.85-2.00)	0.450	531.2
		A/A	20 (10.5%)	18 (9.5%)	1.00 (0.50-2.01)		
	Dominant	T/T	100 (52.6%)	90 (47.4%)	1.00		
		A/T-A/A	90 (47.4%)	100 (52.6%)	1.23 (0.83-1.85)	0.300	529.7
	Recessive	T/T-A/T	170 (89.5%)	172 (90.5%)	1.00		
		A/A	20 (10.5%)	18 (9.5%)	0.89 (0.45-1.74)	0.730	530.7

	Overdominant	T/T-A/A	120 (63.2%)	108 (56.8%)	1.00	0.210	529.2
		A/T	70 (36.8%)	82 (43.2%)	1.30 (0.86-1.96)		
ITGAV	Codominant	C/C	97 (51%)	89 (46.8%)	1.00		
**rs3911238**		G/C	81 (42.6%)	80 (42.1%)	1.08 (0.71-1.64)	0.240	530.0
		G/G	12 (6.3%)	21 (11.1%)	1.91 (0.89-4.10)		
	Dominant	C/C	97 (51%)	89 (46.8%)	1.00		
		G/C-G/G	93 (49%)	101 (53.2%)	1.18 (0.79-1.77)	0.410	530.1
	Recessive	C/C-G/C	178 (93.7%)	169 (89%)	1.00		
		G/G	12 (6.3%)	21 (11.1%)	1.84 (0.88-3.86)	0.099	528.1
	Overdominant	C/C-G/G	109 (57.4%)	110 (57.9%)	1.00	0.920	530.8
		G/C	81 (42.6%)	80 (42.1%)	0.98 (0.65-1.47)		
ITGAV	Codominant	G/G	75 (39.5%)	83 (43.7%)	1.00		
**rs3768777**		A/G	90 (47.4%)	83 (43.7%)	0.83 (0.54-1.28)	0.700	532.1
		A/A	25 (13.2%)	24 (12.6%)	0.87 (0.46-1.65)		
	Dominant	G/G	75 (39.5%)	83 (43.7%)	1.00		
		A/G-A/A	115 (60.5%)	107 (56.3%)	0.84 (0.56-1.26)	0.400	530.1
	Recessive	G/G-A/G	165 (86.8%)	166 (87.4%)	1.00	0.880	530.8
		A/A	25 (13.2%)	24 (12.6%)	0.95 (0.52-1.74)		
	Overdominant	G/G-A/A	100 (52.6%)	107 (56.3%)	1.00	0.470	530.3
		A/G	90 (47.4%)	83 (43.7%)	0.86 (0.58-1.29)		

[a] n, number; [b] OR, odds ratio; [c] 95% CI, confidence interval; [d] *P*-value; [e] AIC, Akaike information criterion; [f] NA, not analyzed.

Categorical variables are presented as numbers (%).

Table S4. Distribution of *HLX* rs868058 genotypes between women with early-onset and late-onset FGR, adjusted by adverse pregnancy outcomes.

Pregnancy disorders	Genetic model	Genotype	Genotype prevalence, n (%) [a]		OR [c] (95 % CI [d])	*P*-value [e]	AIC [f]
			Late-onset FGR [b]	Early-onset FGR			
**Anemia**	Codominant	T/T	69 (53.5%)	21 (36.2%)	1.00		
		A/T	48 (37.2%)	32 (55.2%)	2.45 (1.23-4.90)	**0.034**	223.1
		A/A	12 (9.3%)	5 (8.6%)	1.30 (0.39-4.32)		
	Dominant	T/T	69 (53.5%)	21 (36.2%)	1.00	**0.018**	222.3
		A/T-A/A	60 (46.5%)	37 (63.8%)	2.20 (1.13-4.27)		
	Recessive	T/T-A/T	117 (90.7%)	53 (91.4%)	1.00	0.750	227.8
		A/A	12 (9.3%)	5 (8.6%)	0.83 (0.27-2.59)		
	Overdominant	T/T-A/A	81 (62.8%)	26 (44.8%)	1.00	**0.010**	221.3
		A/T	48 (37.2%)	32 (55.2%)	2.35 (1.21-4.54)		
**Asthma and respiratory system infections**	Codominant	T/T	69 (53.5%)	21 (36.2%)	1.00		
		A/T	48 (37.2%)	32 (55.2%)	2.19 (1.13-4.26)	0.064	233.5
		A/A	12 (9.3%)	5 (8.6%)	1.40 (0.44-4.44)		
	Dominant	T/T	69 (53.5%)	21 (36.2%)	1.00	**0.027**	232.1
		A/T-A/A	60 (46.5%)	37 (63.8%)	2.04 (1.07-3.86)		
	Recessive	T/T-A/T	117 (90.7%)	53 (91.4%)	1.00	0.910	236.9
		A/A	12 (9.3%)	5 (8.6%)	0.94 (0.31-2.81)		
	Overdominant	T/T-A/A	81 (62.8%)	26 (44.8%)	1.00	**0.023**	231.8
		A/T	48 (37.2%)	32 (55.2%)	2.07 (1.10-3.89)		
**Bleeding**	Codominant	T/T	69 (53.5%)	21 (36.2%)	1.00		
		A/T	48 (37.2%)	32 (55.2%)	2.17 (1.11-4.21)	0.069	233.9
		A/A	12 (9.3%)	5 (8.6%)	1.35 (0.42-4.28)		
	Dominant	T/T	69 (53.5%)	21 (36.2%)	1.00	**0.031**	232.6
		A/T-A/A	60 (46.5%)	37 (63.8%)	2.00 (1.06-3.80)		

	Recessive	T/T-A/T	117 (90.7%)	53 (91.4%)	1.00	0.860	237.2
		A/A	12 (9.3%)	5 (8.6%)	0.91 (0.30-2.70)		
	Overdominant	T/T-A/A	81 (62.8%)	26 (44.8%)	1.00	**0.024**	232.2
		A/T	48 (37.2%)	32 (55.2%)	2.06 (1.10-3.86)		
**Diabetes mellitus**	Codominant	T/T	69 (53.5%)	21 (36.2%)	1.00	0.061	233.6
		A/T	48 (37.2%)	32 (55.2%)	2.20 (1.14-4.28)		
		A/A	12 (9.3%)	5 (8.6%)	1.38 (0.43-4.37)		
	Dominant	T/T	69 (53.5%)	21 (36.2%)	1.00	**0.027**	232.3
		A/T-A/A	60 (46.5%)	37 (63.8%)	2.04 (1.08-3.86)		
	Recessive	T/T-A/T	117 (90.7%)	53 (91.4%)	1.00	0.890	237.2
		A/A	12 (9.3%)	5 (8.6%)	0.92 (0.31-2.76)		
	Overdominant	T/T-A/A	81 (62.8%)	26 (44.8%)	1.00	**0.021**	231.9
		A/T	48 (37.2%)	32 (55.2%)	2.09 (1.11-3.92)		
**Hypothyroidism**	Codominant	T/T	69 (53.5%)	21 (36.2%)	1.00	0.058	233.7
		A/T	48 (37.2%)	32 (55.2%)	2.22 (1.14-4.33)		
		A/A	12 (9.3%)	5 (8.6%)	1.42 (0.45-4.53)		
	Dominant	T/T	69 (53.5%)	21 (36.2%)	1.00	**0.024**	232.3
		A/T-A/A	60 (46.5%)	37 (63.8%)	2.07 (1.09-3.93)		
	Recessive	T/T-A/T	117 (90.7%)	53 (91.4%)	1.00	0.910	237.4
		A/A	12 (9.3%)	5 (8.6%)	0.94 (0.31-2.81)		
	Overdominant	T/T-A/A	81 (62.8%)	26 (44.8%)	1.00	**0.021**	232.1
		A/T	48 (37.2%)	32 (55.2%)	2.10 (1.12-3.93)		
**Threatened miscarriage**	Codominant	T/T	69 (53.5%)	21 (36.2%)	1.00	0.062	234.0
		A/T	48 (37.2%)	32 (55.2%)	2.20 (1.13-4.27)		
		A/A	12 (9.3%)	5 (8.6%)	1.37 (0.43-4.33)		
	Dominant	T/T	69 (53.5%)	21 (36.2%)	1.00	**0.027**	232.7
		A/T-A/A	60 (46.5%)	37 (63.8%)	2.03 (1.07-3.85)		

	Recessive	T/T-A/T	117 (90.7%)	53 (91.4%)	1.00	0.880	237.5
		A/A	12 (9.3%)	5 (8.6%)	0.92 (0.31-2.74)		
	Overdominant	T/T-A/A	81 (62.8%)	26 (44.8%)	1.00	**0.022**	232.3
		A/T	48 (37.2%)	32 (55.2%)	2.09 (1.11-3.91)		
**Thrombocytopenia**	Codominant	T/T	67 (53.6%)	20 (35.1%)	1.00	0.064	226.4
		A/T	46 (36.8%)	32 (56.1%)	2.23 (1.13-4.39)		
		A/A	12 (9.6%)	5 (8.8%)	1.35 (0.42-4.32)		
	Dominant	T/T	67 (53.6%)	20 (35.1%)	1.00	**0.030**	225.2
		A/T-A/A	58 (46.4%)	37 (64.9%)	2.04 (1.06-3.93)		
	Recessive	T/T-A/T	113 (90.4%)	52 (91.2%)	1.00	0.840	229.9
		A/A	12 (9.6%)	5 (8.8%)	0.89 (0.30-2.69)		
	Overdominant	T/T-A/A	79 (63.2%)	25 (43.9%)	1.00	**0.022**	224.6
		A/T	46 (36.8%)	32 (56.1%)	2.11 (1.11-4.01)		
**Urogenital infections**	Codominant	T/T	69 (53.5%)	21 (36.2%)	1.00	0.058	233.7
		A/T	48 (37.2%)	32 (55.2%)	2.23 (1.14-4.33)		
		A/A	12 (9.3%)	5 (8.6%)	1.36 (0.43-4.30)		
	Dominant	T/T	69 (53.5%)	21 (36.2%)	1.00	**0.026**	232.5
		A/T-A/A	60 (46.5%)	37 (63.8%)	2.05 (1.08-3.88)		
	Recessive	T/T-A/T	117 (90.7%)	53 (91.4%)	1.00	0.860	237.4
		A/A	12 (9.3%)	5 (8.6%)	0.91 (0.30-2.72)		
	Overdominant	T/T-A/A	81 (62.8%)	26 (44.8%)	1.00	**0.020**	232.0
		A/T	48 (37.2%)	32 (55.2%)	2.11 (1.12-3.98)		

[a] n, number; [b] FGR, fetal growth restriction; [c] OR, odds ratio; [d] 95% CI, confidence interval; [e] *P*-value, statistically significant results are marked in bold;
[f] AIC, Akaike information criterion.

Categorical variables are presented as numbers (%).

Table S5. Distribution of the alleles from the *ANGPT2*, *DLX3*, *HLX*, and *ITGAV* SNPs between women with fetal growth restriction and healthy controls.

Polymorphism	Allele	No. [a] of alleles (%)		Chi-square	*P*-value [b]	No. of alleles (%)	Chi-square	*P*-value
		Controls	FGR [c] cases			Early-onset FGR cases		
ANGPT2 **-35 G>C**	C	24 (6.3%)	25 (6.6%)	0.022	0.883	8 (6.9%)	0.050	0.824
	G	356 (93.7%)	355 (93.4%)			108 (93.1%)		
DLX3 **rs11656951**	A	62 (16.3%)	60 (15.8%)	0.039	0.843	18 (15.5%)	0.042	0.838
	G	318 (83.7%)	320 (84.2%)			98 (84.5%)		
**rs2278163**	C	308 (81.1%)	312 (82.1%)	0.140	0.708	94 (81.0%)	≤ 0.001	0.997
	T	72 (18.9%)	68 (17.9%)			22 (19.0%)		
**rs10459948**	A	23 (6.1%)	19 (5.0%)	0.403	0.525	6 (5.2%)	0.125	0.724
	C	357 (93.9%)	361 (95.0%)			110 (94.8%)		
HLX **rs2184658**	C	289 (76.1%)	299 (78.7%)	0.751	0.386	95 (81.9%)	1.736	0.188
	G	91 (23.9%)	81 (21.3%)			21 (18.1%)		
**rs868058**	A	110 (28.9%)	118 (31.1%)	0.401	0.527	42 (36.2%)	2.204	0.138
	T	270 (71.1%)	262 (68.9%)			74 (63.8%)		
ITGAV **rs3911238**	C	275 (72.4%)	258 (67.9%)	1.815	0.178	83 (71.6%)	0.030	0.864
	G	105 (27.6%)	122 (32.1%)			33 (28.4%)		
**rs3768777**	A	140 (36.8%)	131 (34.5%)	0.465	0.496	42 (36.2%)	0.015	0.901
	G	240 (63.2%)	249 (65.5%)			74 (63.8%)		

[a] No., number; [b] *P*-value; [c] FGR, fetal growth restriction.

Categorical variables are presented as numbers (%)

Table S6. Distribution of the alleles from the *ANGPT2*, *DLX3*, *HLX*, and *ITGAV* polymorphisms between women with early and late-onset FGR.

Polymorphism	Allele	No. [a] of alleles (%)		Chi-square	*P*-value [b]
		Late-onset FGR [c]	Early-onset FGR		
ANGPT2					
-35 G>C	C	16 (6.2%)	8 (6.9%)	0.064	0.800
	G	242 (93.8%)	108 (93.1%)		
DLX3					
rs11656951	A	40 (15.5%)	18 (15.5%)	≤ 0.001	0.997
	G	218 (84.5%)	98 (84.5%)		
rs2278163	C	215 (83.3%)	94 (81.0%)	0.295	0.587
	T	43 (16.7%)	22 (19.0%)		
rs10459948	A	12 (4.7%)	6 (5.2%)	0.047	0.828
	C	246 (95.3%)	110 (94.8%)		
HLX					
rs2184658	C	198 (76.7%)	95 (81.9%)	1.252	0.263
	G	60 (23.3%)	21 (18.1%)		
rs868058	A	72 (27.9%)	42 (36.2%)	2.601	0.107
	T	186 (72.1%)	74 (63.8%)		
ITGAV					
rs3911238	C	174 (67.4%)	83 (71.6%)	0.629	0.428
	G	84 (32.6%)	33 (28.4%)		
rs3768777	A	89 (34.5%)	42 (36.2%)	0.103	0.748
	G	169 (65.5%)	74 (63.8%)		

[a] No., number; [b] *P*-value; [c] FGR, fetal growth restriction.

Categorical variables are presented as numbers (%).
